# Determinants associated with high-risk fertility behaviours among reproductive aged women in Bangladesh: a cross-sectional study

**DOI:** 10.1186/s12978-022-01333-w

**Published:** 2022-01-21

**Authors:** Md. Hasan Howlader, Harun Or Roshid, Satyajit Kundu, Henry Ratul Halder, Sanjoy Kumar Chanda, Md. Ashfikur Rahman

**Affiliations:** 1 Development Studies Discipline, Khulna University, Khulna, 9208 Bangladesh; 2 Statistics Discipline, Khulna University, Khulna, 9208 Bangladesh; 3 School of Public Health, Southeast University, Nanjing, 210009 China; 4 Faculty of Nutrition and Food Science, Patuakhali Science and Technology University, Dumki, Patuakhali, 8602 Bangladesh; 5 Department of Community Health Sciences, Max Rady College of Medicine, University of Manitoba, Winnipeg, MB Canada; 6 Sociology Discipline, Khulna University, Khulna, 9208 Bangladesh; 7 Development Studies Discipline, Khulna University, Khulna, 9208 Bangladesh

**Keywords:** High-risk fertility behaviour, Reproductive age, Women, Determinants, Bangladesh

## Abstract

**Background:**

We aimed to determine the factors that increase the risk of HRFB in Bangladeshi women of reproductive age 15–49 years.

**Methods:**

The study utilised the latest Bangladesh Demographic and Health Survey (BDHS) 2017–18 dataset. The Pearson's chi-square test was performed to determine the relationships between the outcome and the independent variables, while multivariate logistic regression analysis was used to identify the potential determinants associated with HRFB.

**Results:**

Overall 67.7% women had HRFB among them 45.6% were at single risk and 22.1% were at multiple high-risks. Women’s age (35–49 years: AOR = 6.42 95% CI 3.95–10.42), who were Muslims(AOR = 5.52, 95% CI 2.25–13.52), having normal childbirth (AOR = 1.47, 95% CI 1.22–1.69), having unwanted pregnancy (AOR = 10.79, 95% CI 5.67–18.64) and not using any contraceptive methods  (AOR = 1.37, 95% CI 1.24–1.81) were significantly associated with increasing risk of having HRFB. Alternatively, women and their partners’ higher education were associated with reducing HRFB.

**Conclusion:**

A significant proportion of Bangladeshi women had high-risk fertility behaviour which is quite alarming. Therefore, the public health policy makers in Bangladesh should emphasis on this issue and design appropriate interventions to reduce the maternal HRFB.

## Plain Language Summary

High rates of maternal high-risk fertility behaviour (HRFB) have a variety of unfavourable repercussions for both the mother and the child. However, because there have been few studies on this topic to date, we set out to identify the determinants that enhance the risk of HRFB in Bangladeshi women between the ages of 15 and 49. Using latest demographic and health survey (BDHS) data we have found that 67.7% of women had HRFB, with 45.6% having a single high-risk factor and 22.1% having multiple high-risk factors. This high prevalence rate demonstrates that HRFB are all too common in Bangladesh, potentially endangering the health of the country's women. We found that women practicing Islam as core religion, age above 35 years, having normal childbirth, having above 3 children, having unwanted pregnancies and not using birth control methods were at increased risk of having HRFB. As a result of the study's findings, interventions are urgently needed to prevent high-risk fertility behaviour among Bangladeshi women aged 15 to 49 years.

## Background

Women’s high-risk fertility behaviour (HRFB), which is defined by “narrow birth intervals, high birth order, and younger maternal age at birth, have been associated with negative health outcomes for both the mother and the child” [1, 2]. Maternal HRFB is a bio-demographic risk factor that impedes the achievement of lower maternal and child morbidity and mortality [3–7]. Some demographic variables, such as women’s age, parity, and birth spacing are the crucial parameters of measuring HRBF including too-early (< 18 years) or too-late (> 34 years) childbearing, short birth intervals (< 24 months) and a higher number of live births (4 or higher) [3, 4, 7, 8]. Although the total fertility rate (TFR) of Bangladesh declined from 3.7 in 1995 to 2.04 in 2020 [9]. Remarkably the rate of teenage pregnancy is about 35% and 15·1% gave birth less than 24 months interval in Bangladesh. Comparing with many developing countries Bangladesh has the highest rates of adolescent fertility with 82 births per 1000 women as of 2019 where over 50 percent of adolescents gave birth between the years 15–19 [10].

Several studies identified that early or late motherhood is associated with hypertension, premature labor, anemia, gestational diabetes, diabetes, obesity, pregnancy related complications, higher rates of caesarean and operative deliveries and unsafe abortions [11, 12]. Childbearing at an early age (< 18 years) is connected to a growing risk of intrauterine growth restriction, child undernutrition, preterm birth, and infant mortality. On the other hand, late motherhood (> 34 years) is related to preterm births, intrauterine growth restriction, stillbirths, amniotic fluid embolism, chromosomal abnormalities and low-birth-weight newborns [12, 13]. HRFB in mothers also associated with the neonatal mortality; while a study in India identified causal effect of birth spacing on neonatal mortality [14], and also childbearing at teenage was also found to be linked to neonatal mortality [15].

Some previous studies established a relationship between numerous HRFB-related parameters and their detrimental effects on maternal and infant health [7, 8, 16, 17]. Women who start having children at an early age often have more children [18] and this is also associated with adverse maternal, infant and child health outcomes [19]. One the other hand, short birth intervals (< 24 months) [20] and higher birth order [21] may also aggravate the infant and child mortality. Although such evidence supports the consideration of different exposures to high-risk fertility behaviors as a high-priority maternal and child health concern, very few studies in Bangladesh have evaluated factors related to HRFB in women of reproductive age. Therefore, in order to develop effective prevention programs for the region, a clear understanding of the determinants and potential risk factors for maternal high-risk fertility behavior among Bangladeshi women is required. There is, however, a dearth of literature examining the risk factors for HRFB in Bangladesh. To date, most of the studies on HRFB in Bangladesh focused on identifying the relationship between HRFB in women, and maternal and child health outcome [7, 17, 22]. Based on these considerations, this study aimed to identify the associated factors of HRFB in women. Identifying such determinants will be crucial for formulating evidence-based programs in Bangladesh especially targeting the significant risk factors.

## Methods

### Data sources

The study relied on data from the Bangladesh Demographic and Health Survey 2017–18. The National Institute of Population Research and Training (NIPORT) of the Ministry of Health and Family Welfare of Bangladesh used a two-stage stratified sampling approach to conduct this cross-sectional study. The outcomes of our study were assessed using a total sample of 7757 women (age 15 to 49). The study included ever-married women aged 15–49 who were not pregnant currently and had at least one child before the survey. Unmarried and pregnant mothers with incomplete BMI information were excluded as the sample. The description about the data collection procedures and sampling frame are detailed in the original (BDHS 2017–18) report [23].

### Outcome variable

The outcome variable for this study took into account maternal “high-risk fertility behaviour” developed using the definition of the BDHS [23]. The study considered three variables to define the high-risk-fertility behaviour: (a) maternal age at the time of delivery, (b) birth order, and (c) birth interval. The presence of any of the following conditions was termed as a single high-risk fertility behaviour: (i) mothers age less than 18 years at the time of childbirth (ii) mothers age over 34 years at the time of childbirth (iii) latest child born less than 24 months after the previous birth; and (iv) latest child’s birth order 3 or higher. Multiple high-risk categories are made up of two or more aforesaid conditions. High-risk fertility behaviour was defined as the presence of any of the four conditions listed above (coded as 1 and otherwise 0) for final analysis.

### Independent variables

The researchers reviewed the most recent relevant articles to determine the independent variables. The selected sociodemographic and economic variables (independent) included in the analysis are: place of residence (urban and rural), administrative division (Barishal, Chottogram, Dhaka, Khulna, Mymensingh, Rajshahi, Rangpur, Sylhet), religion (Islam, Hindu and other), age (15–24, 25–34 and 35–49 years), age at marriage (< 18 and ≥ 18 years), education (no education, primary, secondary and higher), access to television (no and yes), body mass index (according to WHO [24]; underweight: < 18.50 kg/m^2^, normal: 18.50–24.99 kg/m^2^, overweight/obese: ≥ 25.00 kg/m^2^), current working status (currently working and not working), partner’s education (no education, primary, secondary, higher); partner’s occupation (agricultural, business, non-agricultural, other). Reproductive factors: birth order (1–2, > 3), antenatal care (ANC) seeking (no, yes) and current use of contraceptive methods (yes, no), types of childbirth (normal, caesarean), place of childbirth (home, facility birth), pregnancy wanted (then, later, no more).

### Statistical analysis

The frequency and percentage of the selected attributes were determined using descriptive statistics. The Pearson's chi-square test was performed to show the association between the outcome variables and the specified independent variables at the bivariate level. Finally, the factors related to “high-risk fertility behaviour” were determined using a logistic regression analysis with significant components (p-values < 0.05) at the multivariate model. These analyses included both unadjusted odds ratios (UORs) and adjusted odds ratios (AORs) along with 95% confidence intervals (CIs). Multicollinearity among covariates was checked for all models using variance inflation factors (VIFs), which were determined to be the modest with VIF ≤ 2 for all covariates. Statistical package for social sciences (SPSS. 25.0) was used to conduct all statistical analyses.

### Ethical consideration

DHS data is available in the public domain and is freely available to anyone who makes a reasonable request. The entire study protocol was approved by the Bangladesh Ethics Committee and ICF International; thus, we did not need any additional ethical approval. The BDHS 2017–18 report contains details about the ethical approval [23].

## Results

### Background characteristics and prevalence of HRFB

The final study included 7757 women who had given birth within the previous five years. The median (IQR) age of the respondents was 25.0 years (25.0–75.0). More than half (56.8%) of the women aged 15 to 24 years. Most women (71.6%) lived in rural areas, and overwhelmingly large number (90.5%) of them were Muslims. Over half of the women finished secondary education and 62.8% were unemployed (Table 1).

**Table 1:** Background characteristics and bivariate distribution of high-risk fertility behaviour of study participants across different socio-economic variables (n = 7757)

Variables	High-risk fertility problem		Total (%)	Chi-square value	P-value
	No	Yes			
Administrative division				63.463	< 0.001
Barishal	126 (38.8)	199 (61.2)	325 (5.4)		
Chottogram	445 (37.2)	752 (62.8)	1197 (19.9)		
Dhaka	737 (47.5)	816 (52.5)	1553 (25.9)		
Khulna	238 (39.2)	369 (60.8)	607 (9.4)		
Mymensingh	196 (40.4)	289 (59.6)	485 (8.1)		
Rajshahi	257 (35.4)	468 (64.6)	725 (12.1)		
Rangpur	219 (34.2)	422 (65.8)	641 (10.7)		
Sylhet	219 (46.8)	249 (53.2)	468 (7.8)		
Place of residence				39.870	< 0.001
Urban	800 (47.0)	903 (53.0)	1703 (28.4)		
Rural	1637 (38.1)	2660 (61.9)	4297 (71.6)		
Religion				40.736	< 0.001
Islam	2134 (39.3)	3294 (60.7)	5428 (90.5)		
Hindu	275 (52.8)	246 (47.2)	521 (8.7)		
Other	29 (55.8)	23 (44.2)	52 (0.9)		
Maternal age				331.40	< 0.001
15–24	1117 (32.8)	2292 (67.2)	3409 (56.8)		
25–34	1247 (55.2)	1014 (44.8)	2261 (37.7)		
35–49	74 (22.3)	258 (77.7)	332 (5.5)		
Maternal BMI (kg/m^2^)				66.01	< 0.001
Underweight (≤ 18.50)	290 (35.0)	538 (65.0)	828 (13.8)		
Normal (18.51–25.00)	1382 (38.2)	2232 (61.8)	3614 (60.2)		
Overweight/Obese (> 25.00)	765 (49.1)	792 (50.9)	1557 (26.0)		
Maternal education level				527.17	< 0.001
No education	76 (27.8)	197 (72.2)	273 (4.6)		
Primary	441 (29.7)	1043 (70.3)	1484 (24.7)		
Secondary	1117 (36.1)	1977 (63.9)	3094 (51.6)		
Higher	803 (69.9)	345 (30.1)	1148 (10.1)		
Current working status				25.13	< 0.001
Not Working	1623 (43.1)	2145 (56.9)	3768 (62.8)		
Working	815 (36.5)	1418 (63.5)	2233 (37.2)		
Husband’s education level				295.74	< 0.001
No education	209 (29.7)	495 (50.3)	704 (11.7)		
Primary	651 (33.4)	1298 (66.6)	1949 (32.5)		
Secondary	826 (39.0)	1293 (61.0)	2119 (35.3)		
Higher	752 (61.2)	476 (38.8)	1228 (20.5)		
Husband’s occupation				58.57	< 0.001
Agriculture	350 (32.7)	720 (67.3)	1070 (17.8)		
Business	573 (44.7)	710 (55.3)	1283 (21.4)		
Service	1285 (43.3)	1682 (56.7)	2967 (49.4)		
Other	230 (33.8)	451 (66.2)	681 (11.3)		
Wealth status				207.02	< 0.001
Poor	696 (30.4)	1591 (69.6)	2287 (38.1)		
Middle	462 (38.8)	729 (61.2)	1191 (19.8)		
Rich	1280 (50.7)	1243 (49.3)	2523 (42.0)		
Watching TV				75.25	< 0.001
No	658 (32.8)	1342 (67.2)	1998 (33.3)		
Yes	1781 (44.5)	2221 (55.5)	4002 (66.7)		
Sex of the child				0.99	0.332
Male	1289 (41.2)	1838 (58.8)	3127 (52.1)		
Female	1148 (40.0)	1725 (60.0)	2873 (47.9)		
Types of toilet use				65.30	< 0.001
Unimproved	472 (31.7)	1017 (68.3)	1489 (24.8)		
Improved	1965 (43.6)	2546 (42.4)	4511 (75.2)		

**Fig. 1:**
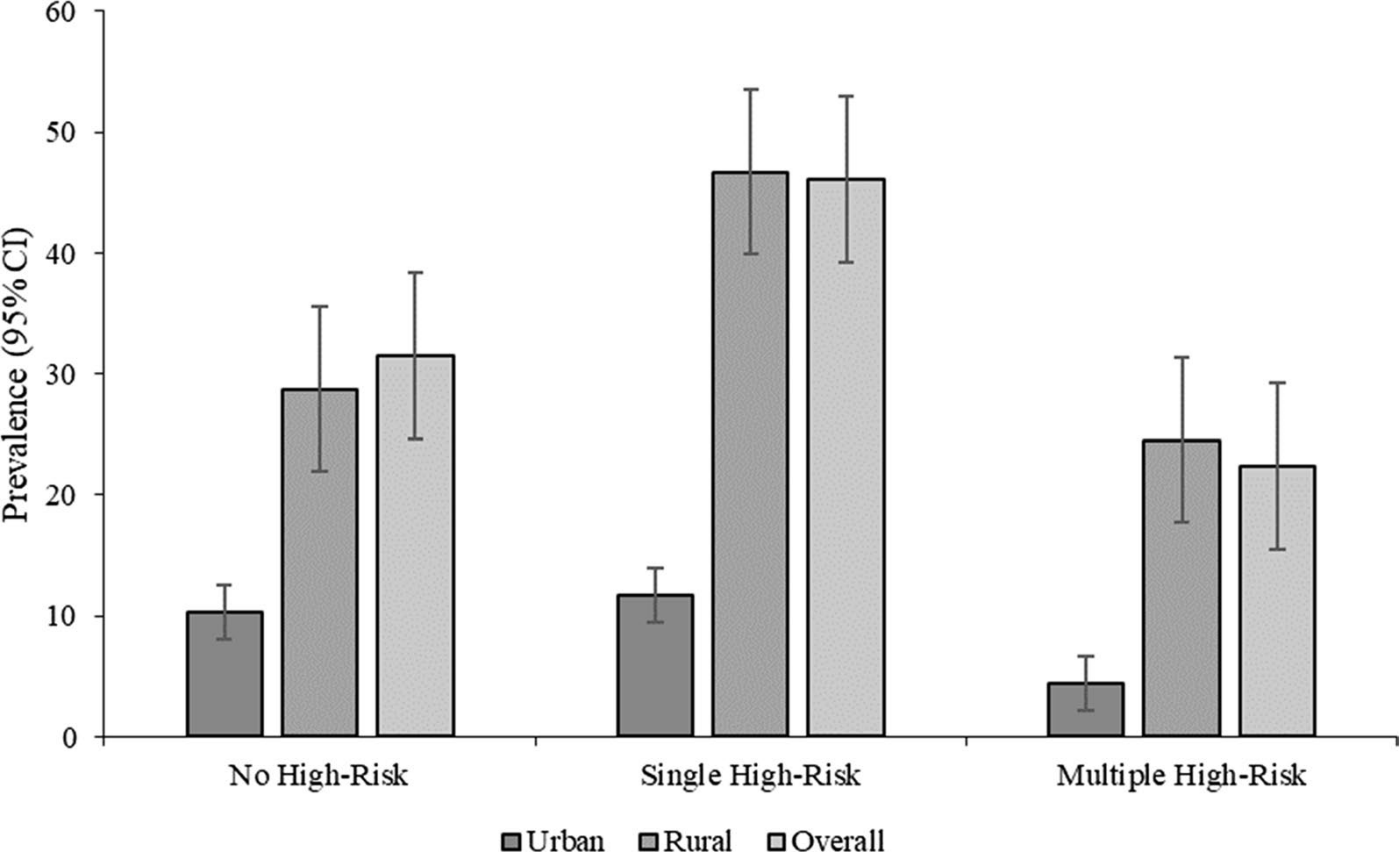
Prevalence of HRFB by place of residence

**Fig. 2:**
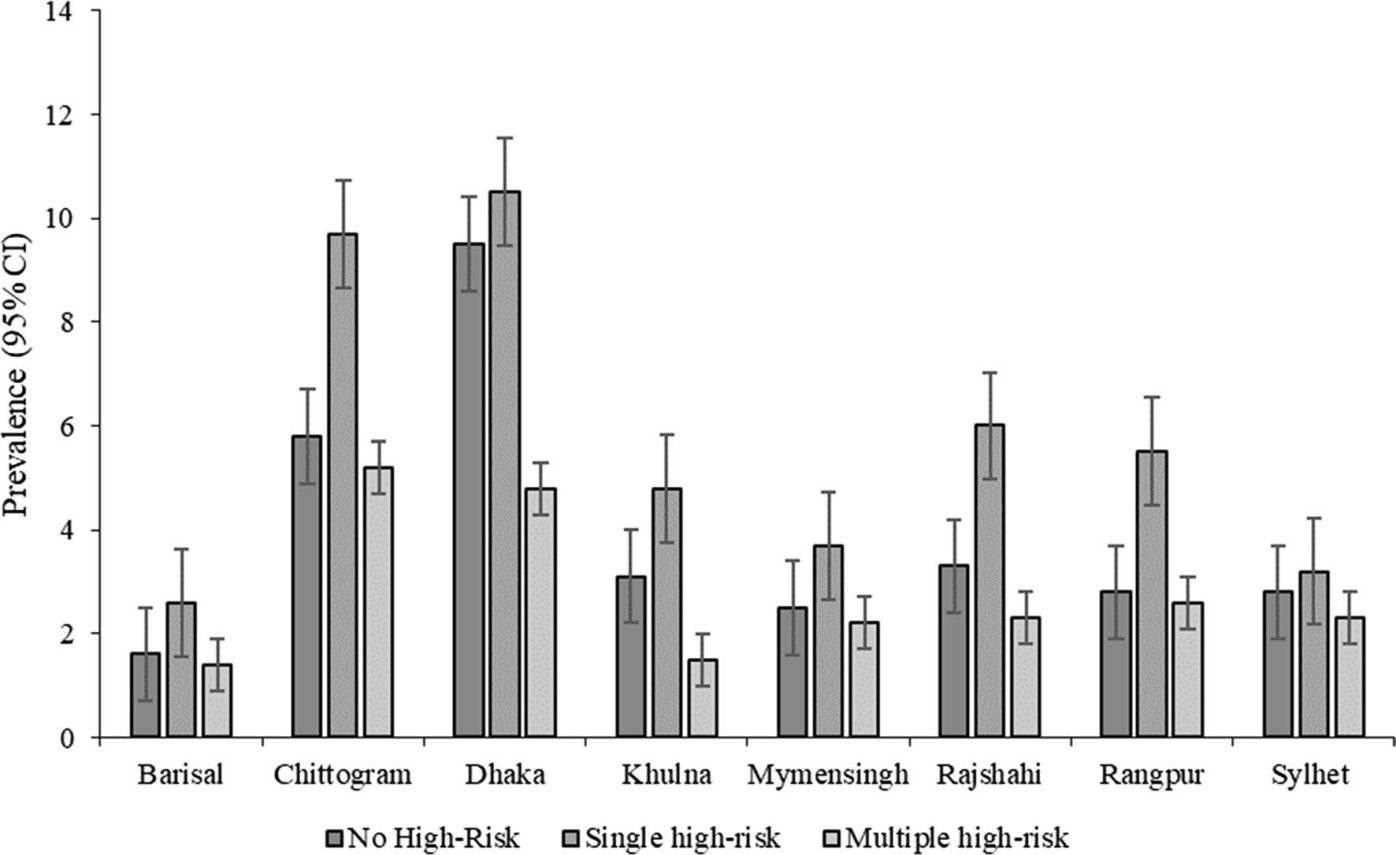
Prevalence of HRFB by administrative regions

The worst situation was found in the rural areas for both the single and multiple HRFB. About 46.7% of respondents from rural areas had single HRFB compared to 11.7% from urban areas. Similarly, 24.5% of women from rural areas were at multiple HRFB, which was only 4.4% among women from urban areas (Fig. 1). Figure 2 demonstrates the prevalence of HRFB across different administrative divisions of Bangladesh. The highest prevalence of single risk fertility behaviour was found in Dhaka (10.5%), followed by Chottogram division (9.7%). However, the highest rate of multiple HRFB was found in Chottogram division.

### Reproductive characteristics and high-risk fertility behaviour

Most women (63.8%) have had a recent normal childbirth and 54.3% have given birth at a healthcare center. Of the total mothers, a significant portion (91.9%) completed ANC follow-up for their recent pregnancy (Table 2).

**Table 2:** Bivariate distribution of high-risk fertility behaviour of respondents across different reproductive factors (n = 7757)

Variables	High-risk fertility		Total (%)	Chi-square value	P-Value
	No	Yes			
Place of delivery				128.14	< 0.001
Facility Birth	1056 (51.4)	999 (48.6)	2055 (54.3)		
Home	573 (33.1)	1158 (66.9)	1731 (45.7)		
Type of delivery				136.005	< 0.001
Normal	952 (35.3)	1747 (64.7)	2699 (63.8)		
Caesarean	821 (53.7)	708 (46.3)	1529 (36.2)		
ANC seeking (n = 4605)				27.47	< 0.001
No	61 (26.8)	167 (73.2)	228 (8.1)		
Yes	1535 (44.5)	1913 (55.5)	3448 (91.9)		
Pregnancy wanted (n = 4814)				61.11	< 0.001
Then	1431 (45.2)	1732 (54.8)	3163 (83.6)		
Later	185 (35.9)	330 (64.1)	515 (13.6)		
No more	12 (11.2)	95 (88.8)	107 (2.8)		
Contraceptive use				11.34	< 0.001
Yes	1596 (39.2)	2479 (60.8)	4075 (67.9)		
No	842 (43.2)	1083 (56.3)	1925 (32.1)		

### Factors associated with high-risk fertility behaviour

Both univariate and multivariate logistic regression models were used to identify potential risk variables, however because this model was controlled for confounding effects of covariates, we only used the adjusted results to interpret the findings. The women who were Muslims having higher risk of Fertility behavior (Adjusted Odds Ratio [AOR] = 5.52, 95% Confidence Interval [CI] 2.25–13.52, p < 0.001) than that of other religion. HRFB was found 19% less common in younger women (15–24 years; AOR = 0.19, 95% CI 0.10–0.30, p < 0.001) and 6.42 times more likely in women over 35 years (AOR = 6.42 95% CI 3.95–10.42, p < 0.001). Women who had normal childbirths possessed higher HRFB (AOR = 1.47, 95% CI 1.22–1.69, p = 0.003) compared to those who had a caesarean section. Women who had unwanted pregnancies were 10.79 times more likely to have high-risk fertility than women whose pregnancies were desired (AOR = 10.79, 95% CI 5.67–18.64, p < 0.001). Women who did not presently use contraceptive methods were 1.37 times more likely to have HRFB compared to their counterparts (AOR = 1.37, 95% CI 1.24–1.81, p < 0.001). The odds of HRFB disproportionately distributed across the divisional regions. On the other hand, women aged 25 to 34, having secondary and higher education level; partner’s higher-level education reduced the odds of high HRFB (Table 3).

**Table 3:** Factors associated with high-risk fertility behaviour among women: BDHS 2017–18

	UOR (95% CI)	P-value	AOR (95 CI)	P-value
Administrative regions				
Barishal	1.20 (0.99–1.46)	0.062	1.88 (1.29–2.75)	0.001
Chottogram	1.29 (1.09–1.53)	0.003	2.17 (1.63–2.89)	< 0.001
Dhaka	0.83 (0.70–0.98)	0.027	1.27 (0.96–1.68)	0.094
Khulna	0.98 (0.81–1.18)	0.842	2.11 (1.51–2.95)	< 0.001
Mymensingh	1.22 (1.01–1.47)	0.037	1.55 (1.11–2.17)	0.010
Rajshahi	1.25 (1.03–1.56)	0.022	1.91 (1.39–2.63)	< 0.001
Rangpur	1.36 (1.23–1.65)	0.001	2.22 (1.60–3.09)	< 0.001
Sylhet (Ref.)	1		1	
Place of residence				
Urban (Ref.)	1		1	
Rural	1.59 (1.44–1.76)	< 0.001	0.87 (0.73–1.03)	0.101
Religion				
Islam	2.23 (1.25–3.98)	0.007	5.52 (2.25–13.52)	< 0.001
Hindu	1.14 (0.63–2.08)	0.660	2.50 (1.90-6.30)	0.053
Other (Ref.)	1		1	
Maternal age				
25–34 (Ref.)	1		1	
15–24	1.29 (1.17–1.42)	< 0.001	0.19 (0.10–0.30)	< 0.001
35–49	3.79 (2.94–4.88)	< 0.001	6.42 (3.95–10.42)	< 0.001
Maternal BMI (kg/m^2^)				
Underweight (≤ 18.50) (Ref.)	1		1	
Normal (18.51–25.00)	0.90 (0.78–1.04)	0.156	1.05 (0.83–1.35)	0.652
Overweight/Obese (≥ 25.00)	0.61 (0.52–0.71)	< 0.001	1.03 (0.87–1.23)	0.730
Maternal education level				
No education (Ref.)	1		1	
Primary	0.77 (0.60–0.99)	0.041	7.05 (4.68–10.63)	< 0.001
Secondary	0.80 (0.36–0.58)	< 0.001	5.85 (4.52–7.581)	< 0.001
Higher	0.90 (0.44–0.98)	< 0.001	0.93 (0.80–1.08)	0.328
Current working status				
Not Working (Ref.)	1		1	
Working	1.48 (1.34–1.63)	< 0.001	0.99 (0.85–1.16)	0.908
Husbands education level				
No education (Ref.)	1		1	
Primary	0.71 (0.60–0.85)	< 0.001	1.59 (1.17–2.15)	0.003
Secondary	0.46 (0.39–0.54)	< 0.001	1.31 (1.04–1.66)	0.022
Higher	0.16 (0.13–0.19)	< 0.001	0.39 (0.12–0.72)	0.001
Husband occupation				
Agriculture (Ref.)	1		1	
Business	0.53 (0.45–0.62)	< 0.001	1.02 (0.78–1.30)	0.894
Service	0.46 (0.40–0.53)	< 0.001	1.01 (0.78–1.30)	0.965
Other	0.89 (0.73–1.08)	0.230	1.02 (0.80–1.22)	0.791
Wealth status				
Poor	1.69 (1.47–1.95)	< 0.001	1.07 (0.85–1.31)	0.538
Middle (Ref.)	1		1	
Rich	0.57 (0.50–0.65)	< 0.001	1.01 (0.83–1.23)	0.938
Watching TV				
No (Ref.)	1		1	
Yes	0.51(0.46–0.56)	< 0.001	0.89(0.78–1.07)	0.076
Types of toilet use				
Unimproved (Ref.)	1		1	
Improved	0.47 (0.42–0.53)	< 0.001	0.97 (0.89–1.24)	0.561
Place of delivery				
Home	0.38 (0.33–0.42)	< 0.001	0.78 (0.72–1.17)	0.104
Facility Birth (Ref.)	1		1	
Type of delivery				
Normal	2.71 (2.41–3.06)	< 0.001	1.47 (1.22–1.69)	0.003
Caesarean (Ref.)	1		1	
ANC seeking				
No (Ref.)	1		1	
Yes	0.34 (0.27–0.45)	< 0.001	0.77 (0.76–1.16)	0.114
Pregnancy wanted (n = 4814)				
Then (Ref.)	1		1	
Later	1.44 (1.20–1.74)	< 0.001	1.26 (0.94–1.65)	0.292
No More	16.29 (9.52–27.89)	< 0.001	10.79 (5.67–18.64)	< 0.001
Contraceptive use				
No	1.24 (1.12–1.38)	< 0.001	1.37 (1.24–1.81)	< 0.001
Yes (Ref.)	1		1	

## Discussion

This study showed that 67.7% of women had HRFB, of which 45.6% were in single high-risk category and 22.1% women have had multiple high-risk categories. This high prevalence rate demonstrates that HRFB are all too common in Bangladesh, potentially endangering the health of the country's women. We found that women who were Muslims, age above 35 years, having normal childbirth, having low literacy level, having unwanted pregnancies, not using birth control methods were at increased risk of having HRFB.

When compared to women who have never had any formal education, those with a higher level of education had a lower likelihood of high-risk fertility behaviour. This result was supported by the previously conducted studies [22, 25–27]. The reason for this could be having no formal education impacts on work status and leads to lower income and independence all of which affect purchasing power, taking proper care of mother during pregnancy, visiting ANC. Women who receive formal education had better knowledge and awareness about self-health consciousness, proper diet and thus lead to low odds of HRFB.

In this study, visiting ANC was found to be facilitating factor for reducing the odds of high HRFB. This is probably due to the fact that antenatal care provides opportunities to reach pregnant women with a variety of interventions that may be essential to their health and well-being [28, 29], thus they were more likely to receive information regarding importance of routine check-up, maternal nutrition, delivery complications and risk of having HRFB. On the other hand, women, who did not have ANC follow-ups for their recent children, had more probability to engage in risky reproductive behaviours. Family planning for extending the time between births was discussed during postnatal care counseling. As a result, decreased ANC seeking during pregnancy may have a role in HRFB.

Another important finding from this study is women who had a history of caesarean delivery were less likely to have high-risk fertility behavior. There are some other studies related to the association between type of delivery and subsequent fertility [30, 31] which have similar results. The reason behind this may be women who have their babies by cesarean section were less likely to have more children than women who have their babies vaginally and also cesarean section delivery was followed by a higher likelihood of actively contraception after that birth, which may lead to low odds of HRFB.

This study revealed that, HRFB was more likely to occur among women who had never taken contraception compared to those who used which is in line with previously did studies elsewhere [32, 33]. One of the goals of contraception is to increase the birth interval and reduce unplanned pregnancies. Women who had unwanted pregnancies were more likely to engage in high-risk reproductive behaviour than those who had previous desired pregnancies. It may be the result of not using contraceptive methods by the women who experienced unwanted pregnancies. This result also corroborates with a study conducted in Nigeria [25].

Moreover, religious belief also did affect maternal HRFB. Our study revealed that the women who were Muslims, have increased odds of HRFB compared with other religious believers. This finding was in line with an Indian study [34], where the author argued that Muslim women are less willing to use contraceptive methods, family planning and they prefer temporary methods over sterilisation, these could be plausible reasons why Muslim women in Bangladesh were at higher risk of having HRFB.

Evidence suggests that maternal age of 35–49 have the higher odds of HRFB than their counterparts. Similar result was found in other study where the author concluded that pregnancy at later stage is associated with significant increases in maternal risks and complications [35, 36] which leads to adverse outcome for both the mother and the child.

Furthermore, high-risk fertility behaviours were found more than double among women in Rangpur, a northern region in Bangladesh, compared to the women who live in Sylhet. This is probably due to the fact that women in remote locations may stay behind in terms of utilizing reproductive health services, such as ANC, poor family planning adopted rates related to religious beliefs and community attitudes, as well as having poor literacy levels. However, this inequity in utilizing reproductive health facilities among different regions in Bangladesh should be minimize to reduce the odds of HRFB. This analysis may lead to important inferences that may help to lower maternal high-risk fertility behaviour and can be useful and relevant in areas where HRFB is ubiquitous. The strengths and limitations of this study have been well-recognised. The study employed the recently published BDHS 2017–18 data which had a large country representative sample size, allowing the findings to be more generalisable. Moreover, appropriate statistical technique applied in the analysis can be used to find probable components and their relationships. However, the study has some limitations. For instance, due to cross-sectional data, outcomes and predictors variables were collected at a point of time; therefore, causality cannot be established. In addition, some important factors, such as dietary factors, physical activity and maternal comorbidity histories are not taken into consideration due to unavailability in the original dataset, but these factors may have been associated with HRFB.

## Conclusions

This study highlighted the pervasiveness of maternal high-risk fertility behaviour among Bangladeshi reproductive aged women. Several significant protective factors, such as maternal and partners’ higher education were associated with lower HRFB. In contrast, being Muslims, age 35 to 49 years, having normal childbirth, having unwanted pregnancies, and not using any birth control tools may increase risk of having HRFB for women. Thus, findings from the study identify the need to develop an intervention; especially focusing on Bangladeshi Muslim women aged 35–49 years to reduce high-risk fertility behaviour. Furthermore, the government of Bangladesh and stakeholders (e.g., NGOs, INGOs) should work jointly to prevent early marriage of women and to enhance awareness and proper education to reduce the high-risk fertility behaviour.

## Data Availability

This study used publicly available Demographic and Health Surveys Program datasets from Bangladesh which can be freely obtained from https://dhsprogram.com/. As a third-party user we don’t have permission to share the data publicly in any platforms.
